# *Escherichia coli* Mono-Association Modulates Ionotropic Receptor-Dependent Behaviors in *Drosophila melanogaster*

**DOI:** 10.3390/insects17030275

**Published:** 2026-03-03

**Authors:** Hazem Al Darwish, Mia Cacao, Tia Hart, Deep Patel, Sammi Russo, Safiyah Salama, Muqaddasa Tariq, Aina T. Ananda, Jennifer S. Sun

**Affiliations:** 1 Department of Biochemistry and Microbiology, Rutgers University, 76 Lipman Drive, New Brunswick, NJ 08901, USA; 2 Department of Biology, Monmouth University, 400 Cedar Avenue, West Long Branch, NJ 07764, USA; tandrian@monmouth.edu

**Keywords:** microbiome, *Escherichia coli*, chemosensation, chemoreceptors, olfaction, behavior, *Drosophila melanogaster*, chemical ecology

## Abstract

Chemosensory systems are crucial for insect survival, enabling host-seeking, food acquisition, and oviposition site selection. While insect-associated microbes are known to influence host development and immunity, their role in modulating chemosensory behavior remains poorly understood. Here, we show that mono-association with *Escherichia coli* alters sensory-driven behaviors in both larval and adult axenic *Drosophila melanogaster*. In larvae, *E. coli* mono-association altered phototaxis and mechanosensory responses across genotypes, while changes in tunneling and thermosensory behaviors were reduced or absent in ionotropic receptor (IR) co-receptor mutants. In adults, *E. coli* mono-association increased attraction to fermentation cues (apple cider vinegar, ethanol) and enhanced sucrose consumption in wild-type and *Orco*-deficient flies, whereas these effects were reduced or absent in IR co-receptor mutants (*IR25a*^−^ and *IR76b*^−^). Together, these findings indicate that under defined gnotobiotic conditions, *E. coli* exposure alters sensory-driven behavioral outputs relative to axenic controls. Effects are reduced or absent in IR co-receptor mutants, consistent with a role for IR pathways in mediating these behavioral shifts. These findings support a role for microbial cues in shaping insect sensory-driven behaviors and highlight the importance of microbial status in interpreting behavioral phenotypes. This work provides a framework for future studies investigating how microbial signals interact with conserved sensory pathways.

## Simple Summary

Insects depend on their sense of smell and taste to find food, mates, and suitable places to reproduce. These behaviors influence ecological interactions and, in some species, contribute to the spread of insect-borne diseases and agricultural losses. Our work shows that microbial status influences insect sensory behaviors and that mono-association with *Escherichia coli* under controlled conditions alters behavioral outputs in flies. Several of these behavioral effects are reduced or absent in ionotropic receptor (IR) co-receptor mutants, including IR25a and IR76b, suggesting that IR pathways contribute to microbe-dependent behavioral modulation. Our findings further support a role for microbes in shaping insect sensory-driven behaviors. Understanding how bacteria influence insect sensory systems provides insight into host–microbe interactions and establishes a framework for future studies investigating how microbial cues shape insect behavior.

## 1. Introduction

Insects are among the most diverse and abundant animals on Earth, occupying nearly every ecological niche. Their survival and reproduction depend heavily on chemosensory systems—olfactory and gustatory pathways—that enable them to locate hosts, secure food resources, and identify optimal oviposition sites [1,2,3]. These sensory modalities integrate volatile and non-volatile cues, driving behaviors that directly influence insect fitness and population dynamics [4,5,6]. Identifying endogenous and exogenous factors that regulate insect chemosensory systems offers a promising avenue for informing more precise and sustainable strategies in the long term. At the molecular level, these behaviors are encoded by conserved chemosensory receptor families that translate environmental chemical cues into neural activity.

*Drosophila* rely heavily on olfactory cues to locate food and oviposition sites. Insects detect these cues through two major chemosensory receptor families: the heteromeric odorant receptor (OR) complex, composed of ligand-specific tuning ORs that pair with the obligate co-receptor Orco [7,8], and the ionotropic receptor (IR) family, which includes broadly expressed co-receptors such as IR25a, IR8a, and IR76b [9,10]. While OR/Orco signaling primarily mediates detection of esters and alcohols, IR pathways are tuned to acids and amines [11].

IRs are an evolutionarily conserved chemosensory family derived from ionotropic glutamate receptors that contribute broadly to odor, temperature, and humidity sensing in insects [9,10,12,13,14]. IR25a functions as a widely expressed, obligate co-receptor that pairs with multiple tuning IRs and supports detection of acids and amines as well as non-chemical sensory modalities [9]. Consistent with this broad role, loss of IR25a disrupts amine-responsive olfactory neurons and impairs cool-avoidance and hygrosensory behaviors [13,14], establishing IR25a as a central integrator of IR-dependent sensory input. IR76b is another broadly acting IR co-receptor that partners with multiple tuning receptors and plays established roles in gustatory signaling. IR76b contributes to attractive low-salt taste, amino acid sensing, and polyamine detection, thereby influencing nutrient evaluation and feeding behavior [15,16,17,18]. IR76b is expressed across multiple chemosensory neuron classes and participates in IR-dependent coding of ecologically relevant chemicals [19], supporting the idea that IR76b and IR25a represent complementary co-receptor entry points into IR signaling. These properties motivated us to test whether microbial modulation engages IR co-receptor pathways broadly (IR25a and IR76b), rather than reflecting dependence on a single locus.

Several groups have discovered that microbial cues can directly engage olfactory and gustatory circuits to bias insect behavior and influence host physiology, including immune development and metabolic regulation [20,21,22,23,24]. *Drosophila* preferentially orient toward multispecies microbial communities because interspecies metabolite exchange generates emergent volatile cues that reshape olfactory preference and oviposition decisions [25]. For example, in yeast-*Acetobacter* co-cultures, this attraction is largely mediated by the conserved olfactory receptor OR42b and depends on *Acetobacter’s* metabolism of yeast-derived ethanol, with acetate and related metabolites sufficient to reproduce the full community-driven behavioral response [25]. Likewise, microbes can alter taste-guided reproductive choices: commensal enterococci drive oviposition preference for fermented substrates through sucrose catabolism, and this behavioral output requires sucrose-sensing gustatory neurons (GR5a/GR64a), demonstrating that bacterial metabolism can reweight gustatory inputs to steer egg-laying site selection [26]. Microbial signals can also trigger robust avoidance via hardwired olfactory pathways, like how geosmin activates a single OR56a-expressing sensory neuron class and a dedicated downstream circuit that is sufficient and necessary for aversion, suppressing chemotaxis, feeding, and oviposition even in the presence of otherwise attractive food odors [27]. Exposure to pathogen-contaminated media can also suppress larval feeding through olfactory neuronal circuitry via the universal olfactory co-receptor Orco and TrpA1, indicating that bacterial presence can be transduced into acute, neurally mediated behavioral immunity [28]. Bacterial lipopolysaccharide (LPS), a conserved component of Gram-negative cell walls, can also directly activate gustatory neurons via TRPA1 and the bitter receptor Gr66a, triggering aversive feeding responses [29,30].

These studies support the premise that microbial cues can reshape insect sensory-driven behaviors, but the receptor pathways mediating these effects remain incompletely defined. Here, we used a controlled mono-association model to test whether *Escherichia coli* exposure is sufficient to modulate defined larval and adult behaviors in *Drosophila melanogaster* and whether these effects require conserved IR co-receptor signaling through IR25a and IR76b. We used the non-pathogenic laboratory strain *E. coli* K-12 MG1655 (ATCC 47076), which is widely used for experimental manipulation in flies due to its genetic tractability and standardized dosing [22,31,32,33], enabling direct comparisons across conventionally reared, axenic, and *E. coli*-associated cohorts in wild-type, *Orco*^−^, and IR co-receptor mutant backgrounds.

## 2. Materials and Methods

### 2.1. Maintaining Fly Cultures

All experiments used *D. melanogaster* maintained on Nutri-Fly Bloomington Formulation (Genesee Scientific, El Cajon, CA, USA) prepared per manufacturer instructions. Food was autoclaved, cooled to room temperature, then distributed into vials within a biosafety cabinet. Propionic acid (Sigma-Aldrich, St. Louis, MO, USA; final 0.3% *v*/*v*) was added as a fungal inhibitor. Unless otherwise stated, flies were maintained at 25 °C, 60% relative humidity (RH), under an 8 h light/16 h dark (8L:16D) cycle. Populations were age-matched and density-controlled by seeding ~30 adults per vial for oviposition; adults were removed after 24–48 h to standardize larval density. Experimental cohorts were reared and assayed in parallel to minimize batch and seasonal effects. The following lines were used in this study:

+; +; *IR76b*^−^ (RRID:BDSC_51309; abbreviated *IR76b*^−^);

+; *IR25a*^−^; + (RRID:BDSC_41747; abbreviated *IR25a*^−^);

+; +; *Orco*^−^ (RRID:BDSC_23130; abbreviated *Orco*^−^);

*Canton Special* (RRID:BDSC_64349; abbreviated *Canton-S*).

*IR25a*^−^, *Orco*^−^, and *Canton-S* were generously provided by the John R Carlson laboratory at Yale University; *IR76b*^−^ was purchased from the Bloomington Drosophila Stock Center (BDSC). *IR76b*^−^ corresponds to the *IR76b*^1^ allele, a loss-of-function mutation generated by Δ2-3 transposase-mediated P-element mobilization that disrupts the *IR76b* locus and abolishes *IR76b* co-receptor function. *IR25a*^−^ corresponds to the *IR25a*^1^ null allele, which carries a deletion that removes most of the coding sequence and produces a complete loss-of-function. *Orco*^−^ corresponds to the *Orco*^2^ allele, a well-characterized null mutation caused by a P-element insertion disrupting the *Orco* coding region. Prior to experiments, all lines were backcrossed for ten generations to *Canton-S* to standardize genetic background. *D. melanogaster* are invertebrates and are not subject to IACUC oversight under U.S. federal regulations (Animal Welfare Act, 9 CFR Parts 1–3).

### 2.2. Preparation of Axenic (Germ-Free) Flies

The protocol to generate axenic fly cultures by sterilizing embryos was adapted from [34]. Embryos were collected following Cold Spring Harbor Laboratory protocols. Apple-juice agar plates were prepared and spread with 10 µL yeast paste (Bob’s Red Mill, Milwaukie, OR, USA) to stimulate oviposition. Thirty adults were placed on each plate and covered with a sterile beaker to form an oviposition chamber, then incubated for 24 h. Embryos were transferred with a moistened paintbrush to 40 µm mesh cell strainers (Olympus Corporation, Shinjuku City, Tokyo, Japan). Debris was rinsed away with sterile ddH_2_O for 1 min. Embryos were dechorionated in 5% (*v*/*v*) household bleach (The Clorox Company, Oakland, CA, USA) for 5 min, then rinsed thoroughly (3 × 1 min rinses with sterile ddH_2_O). Dechorionated embryos were placed onto fresh, sterile food in autoclaved vials within a biosafety cabinet. Lines were maintained germ-free for 5 generations before use. For verification, we plated homogenates of 10 surface-sterilized adults from axenic cohorts on Luria broth (LB) agar (37 °C, 24–48 h) to confirm absence of colony growth prior to experiments (Figure S1).

qPCR with 16S rRNA also confirmed the absence of bacterial DNA, as described by Clark et al. [35]. Genomic DNA was extracted using the PowerSoil Pro Kit (QIAGEN, Hilden, Germany). Flies were surface-sterilized [36], homogenized in 150 µL of bead solution with a motorized pestle, and processed following the manufacturer’s instructions. qPCR was performed using PowerUp SYBR Green Master Mix (Applied Biosystems, Wilmington, DE, USA) on a StepOnePlus qRT-PCR instrument (Applied Biosystems). Cycling conditions were 95 °C for 10 min, followed by 40 cycles of 95 °C for 15 s and 60 °C for 60 s. Universal 16S primers targeting the V1–V2 region [37] were used to detect total bacterial DNA (V1F: 5′-AGAGTTTGATCCTGGCTCAG-3′; V2R: 5′-CTGCTGCCTYCCGTA-3′). Axenic samples showed no amplification or only late-cycle background Ct values, confirming the absence of detectable bacteria.

### 2.3. Reinfection of Axenic Flies

LB plates were streaked with *E. coli* K-12 carrying empty pET28b (ampR). Isolated colonies were inoculated into LB with ampicillin and grown overnight (16–18 h, 37 °C, 200 rpm). For reinfection, 5 mL overnight culture (OD_600_ = 2.0) was aliquoted into 500 µL volumes, pelleted (e.g., 5000× *g*, 5 min), and each pellet was resuspended in 50 µL LB as concentrated inoculum. This inoculum was spread evenly on the food surface within each sterile fly food vial; a control vial received 50 µL sterile LB only. Axenic adults were flipped into the inoculated vials to oviposit for 7 days, then removed. The resulting progeny (infected generation) were used for assays. Recolonization of axenic flies was validated by plating homogenates of 10 surface-sterilized adults from infected cohorts on LB agar (37 °C, 24–48 h) (Figure S1). qPCR with 16S rRNA primers also confirmed the presence of bacterial DNA. Throughout the manuscript, we use “mono-association” to describe controlled exposure to a single bacterium under gnotobiotic conditions. This terminology avoids implying long-term persistence in the fly, which can vary markedly across bacterial taxa and strains [31].

### 2.4. Environmental Control and Batch Effects

All behavioral assays were performed at 25 °C, 60% RH, in complete darkness unless otherwise stated. Biological replicates were run across ≥2 independent days to distribute potential day effects. To minimize seasonal/batch variability, replicates were run within the same week per condition when possible.

### 2.5. Larval Tunneling Assay

The larval tunneling assay was previously described by Qiang et al. [38]. We prepared 60 mm × 15 mm Petri dishes with 25 mL 0.5% agar. A 1 mm diameter central hole was punched and filled with yeast paste. Ten third-instar larvae were rinsed in sterile ddH_2_O for 1 min with gentle swirling to remove food debris, allowed to acclimate on a separate agar plate for 2 min, then placed into the central well. After 30 min, tunneling trails were imaged; distance (pixels) was quantified with identical exposure and magnification across plates in Fiji version 2.9.0 (RRID:SCR_002285) with a fixed calibration.

### 2.6. Larval Phototaxis Assay

The larval phototaxis assay was previously described by Luna et al. [39]. First, 60 mm × 15 mm Petri dishes with 25 mL 2% agar were prepared. Half the lid and base were occluded with black paint to create light and dark areas. Ten third-instar larvae were briefly rinsed in sterile ddH_2_O with gentle swirling to remove food debris, allowed to acclimate on a separate agar plate for 2 min, placed in the center of the plate, and exposed to constant overhead illumination for 10 min. Preference (%) = (# in dark/total) × 100%.

### 2.7. Larval Temperature Sensitivity Assay

The larval temperature sensitivity assay was previously described by Liu et al. [40]. Ten third-instar larvae were placed in a covered 60 mm × 15 mm Petri dish without agar. For heat, the dish was placed in a water bath held at 32 °C; for cold, at 4 °C; exposure 3 min each. Movement intensity was scored from a video (blinded observer) as distance covered per unit time (pixels/min).

### 2.8. Two-Choice Trap (Olfactory) Assay

Two-choice trap assays were designed with vertically oriented traps suspended below the arena to prevent escape, as previously described by Woodard et al. [41]. Each 1.5 mL Eppendorf tube trap contained 200 µL fly food in the lid, either unmodified (control) or supplemented with 10% ethanol (Sigma) or 10% apple cider vinegar (Heinz, Pittsburgh, PA, USA; ACV). Baited tubes were mounted beneath a small aperture in the arena floor; the aperture was just large enough for a fly to pass, discouraging re-emergence. The vertical orientation ensured once a fly committed, it could not exit easily. Ten virgin adults (5 female, 5 male) were isolated for 2 days post-eclosion and water-starved for 24 h, anesthetized briefly on ice, and introduced to the arena for 24 h. Preference index (PI) = ((# in experimental) − (# in control))/(total in traps) × 100%.

### 2.9. Fly Liquid-Food Interaction Counter (FLIC) Assay

The FLIC system and operational guidance are described by Ro et al. [42]. To assay single-well food interactions, the FLIC system was configured with the *Drosophila* Feeding Monitor (DFM) with single-chamber tops, connected to the Master Control Unit (MCU). Freshly prepared liquid food (5% sucrose in 50 mg/L MgCl_2_; Sigma) was loaded into each channel to form a simple voltage divider when contacted by a fly. Signals were logged and analyzed as a relative feeding signal (arbitrary units) using a custom R script (RRID:SCR_018386). Signals were baseline-corrected. A “lick” was defined as an individual proboscis contact generating a corrected signal exceeding 200 (arbitrary units). An “event” was defined as a continuous sequence of licks separated by no more than 1 s. Event “duration” was calculated as the total time (seconds) from event onset to termination. “Total feeding signal” represents the summed signal intensity across all events per fly during the recording period.

### 2.10. Randomization, Blinding, Inclusion/Exclusion Criteria

Within each genotype and condition, vials were randomized to assay order using a random number generator. Observers were blinded to genotype and infection status for scoring-based assays (temperature, phototaxis) and for image-based quantification (tunneling). Pre-specified exclusion criteria: vials with visible contamination, assays with escaped or immobile flies (>20% of cohort immobile at start), or hardware/electrical faults (FLIC) were discarded and repeated.

### 2.11. Data Analysis

Data were analyzed using two-way ANOVA with genotype and microbial status (conventional, axenic, *E. coli*) as factors, including interaction terms. Where significant interactions were detected, post hoc comparisons were performed using Šídák’s multiple comparisons test to compare microbial conditions within each genotype. With a balanced 4 (genotype) × 3 (microbial status) design and *N* = 20–24 biological replicates per genotype × condition, our study is powered to detect small-to-moderate genotype, microbial status, and genotype × microbial status interaction effects using two-way ANOVA. Šídák’s correction controls for the family-wise error rate across multiple comparisons. Summary plots show mean ± SEM; significance thresholds were * = *p* < 0.05; ** = *p* < 0.01; *** = *p* < 0.001; **** = *p* < 0.0001. Analyses were performed in GraphPad Prism version 11.0.0 (RRID:SCR_002798) or R Project for Statistical Computing version R 4.5.2 (RRID:SCR_001905).

## 3. Results

### 3.1. E. coli Mono-Association Alters Larval Sensory-Driven Behaviors

Insect larvae must integrate short-range sensory inputs—mechanosensation, thermosensation, and phototaxis—to navigate their microhabitats, avoid predation, and secure resources. These modalities are especially critical because larvae, unlike adults, cannot rely on long-distance cues such as odor plumes [1,2,6].

**Figure 1:**
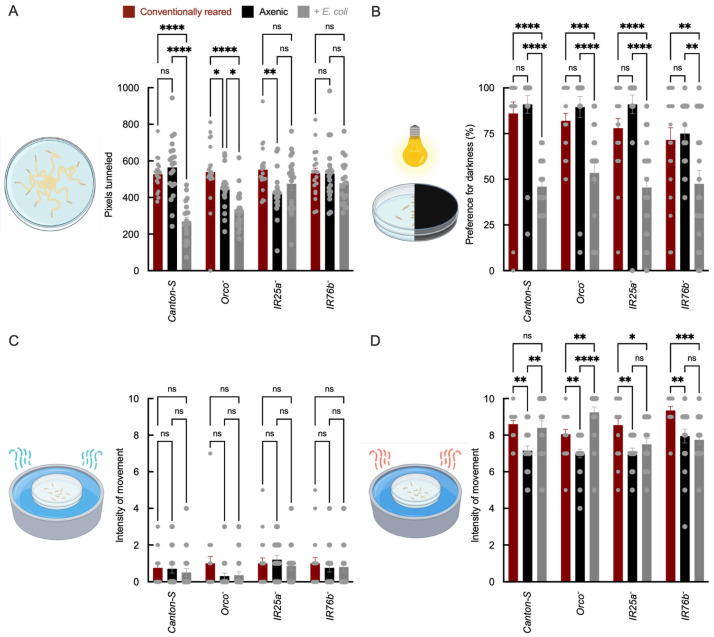
Larval behavioral responses in wild-type and receptor mutant strains under axenic and *E. coli*-recolonized conditions. (**A**) Tunneling distance (y-axis, arbitrary units derived from pixel length) measured as the average distance traveled for 10 larvae per plate; x-axis shows genotype and treatment. (**B**) Phototaxis preference index (y-axis = % larvae in dark zone). (**C**,**D**) Movement intensity (y-axis = pixels/min) in cold (4 °C) and heat (32 °C) assays, measured as the average movement intensity of 10 larvae per plate. Bar colors indicate microbial status: red = conventionally reared, black = axenic, and gray = *E. coli*-associated. For all assays, *N* = 20 plates per genotype × microbial condition (biological replicates). Error bars = mean ± SEM. Statistical comparisons were performed using two-way ANOVA with genotype and microbial status as factors, including the genotype × microbial status interaction term. Planned post hoc comparisons were conducted using Šídák’s multiple comparisons test. Significance: * *p* < 0.05, ** *p* < 0.01, *** *p* < 0.001, **** *p* < 0.0001, ns: not significant.

We observed that larval tunneling distance was reduced in *E. coli*-associated *Canton-S* (wild-type) and *Orco*^−^ larvae relative to conventionally reared controls, whereas IR co-receptor mutants (*IR25a*^−^ and *IR76b*^−^) showed no significant difference between microbial conditions (Figure 1A). Because tunneling behavior can reflect both sensory-driven exploration and broader physiological state (e.g., metabolism or locomotor capacity) [43], these results suggest that microbe-dependent changes in tunneling are attenuated in IR co-receptor mutants, consistent with a contribution of IR pathways to this behavioral output. Microbe-dependent hypoxia and metabolic effects have been reported in other host-microbe systems [44], suggesting that both sensory and systemic pathways may contribute to the observed phenotype.

In phototaxis assays, *E. coli* association reduced negative phototaxis across all genotypes (*Canton-S*, *Orco*^−^, *IR25a*^−^; *IR76b*^−^) in comparison to conventionally reared larvae (Figure 1B), suggesting that this effect is not dependent on the tested chemosensory co-receptors. Because phototaxis can be influenced by general arousal and physiological state [45], this phenotype may reflect systemic consequences of microbial association rather than a receptor-specific sensory mechanism.

By contrast, temperature responsiveness showed IR co-receptor dependence. Relative to axenic larvae, *E. coli*-associated wild-type larvae displayed increased movement intensity in response to heat, whereas this phenotype was reduced or absent in *IR25a*^−^ and *IR76b*^−^ mutants between microbial conditions (Figure 1C,D). IR25a and IR76b are well-established co-receptors for thermal and humidity sensing [13,14,19,46]. These results are consistent with microbial association influencing temperature responsiveness in a manner dependent on IR co-receptor function, although indirect physiological contributions cannot be excluded. The attenuation of microbe-dependent effects in IR co-receptor mutants aligns with evidence that IR pathways contribute to thermosensation and thermoregulatory behavior and complements recent work showing that commensal bacteria can shift larval thermal preference [47,48].

Together, these data show that *E. coli* association alters multiple larval behavioral outputs, including phenotypes that are independent of the tested receptor genotypes (phototaxis and mechanosensation) and phenotypes attenuated in IR co-receptor mutants (tunneling and temperature responsiveness). These results suggest that microbial status influences larval behavior through both IR-dependent and systemic mechanisms.

### 3.2. E. coli Mono-Association Alters Adult Olfactory and Gustatory Behaviors

Adult *Drosophila* rely on long-range olfactory cues and gustatory evaluation to locate and assess nutrient-rich food sources, including fermentation-associated volatiles and sugars that strongly shape foraging behavior [1,2,3].

**Figure 2:**
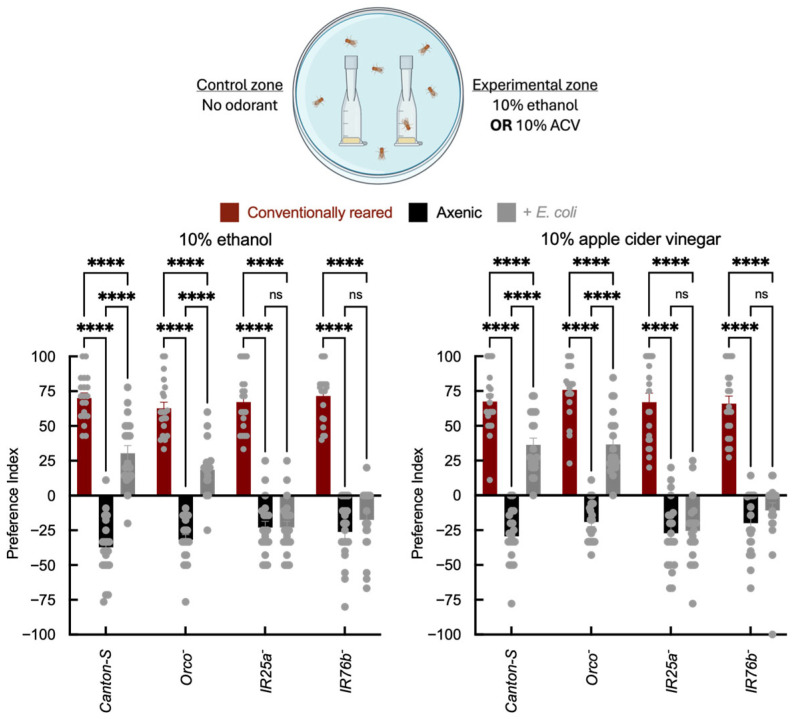
Two-choice olfactory trap assay quantifying adult attraction to fermentation cues. Behavioral assessment of adult wild-type *Canton-S* and receptor mutant strains under axenic conditions and after *E. coli* recolonization. The x-axis indicates genotype (*Canton-S*, *Orco*^−^, *IR25a*^−^, and *IR76b*^−^) and infection status of the insect (axenic vs. *E. coli*). Traps were baited with fly food containing no odorant as a control, or with either 10% ethanol or 10% apple cider vinegar (ACV). The y-axis shows the Preference Index (PI), calculated as ((# in experimental trap) − (# in control trap))/(total in traps) × 100%. Each arena contained 10 adults (5 males, 5 females), and *N* = 20 arenas per genotype × treatment group. Bar colors indicate microbial status: red = conventionally reared, black = axenic, and gray = *E. coli*-associated. Error bars = mean ± SEM. Statistical comparisons were performed using two-way ANOVA with genotype and microbial status as factors, including the genotype × microbial status interaction term. Planned post hoc comparisons were conducted using Šídák’s multiple comparisons test. Significance: **** *p* < 0.0001, ns: not significant.

In two-choice trap assays, conventionally reared *Canton-S* and *Orco*^−^ adults were attracted to ethanol and ACV, whereas axenic flies showed reduced attraction and, in some cases, aversion to these odorants (Figure 2). *E. coli* association partially restored attraction to ethanol and ACV in *Canton-S* and *Orco*^−^ adults relative to axenic flies, although attraction did not reach the level observed in conventionally reared adults. This enhancement was reduced or absent in *IR25a*^−^ and *IR76b*^−^ mutants. These data are consistent with microbial modulation of odor-guided behavior that is attenuated in IR co-receptor mutants under the conditions tested. Vinegar attraction reflects both volatile acids and other fermentation products; importantly, IR25a and IR76b play established roles in acid detection and acid-driven behavioral decisions [17,49], including sour taste and oviposition preference, providing a mechanistic rationale for why microbial status may influence IR-dependent responses to fermentation cues. Because these odorants can be detected by multiple receptor classes, we interpret these results as indicating a contribution from IR-dependent pathways, without excluding additional sensory or physiological mechanisms.

**Figure 3:**
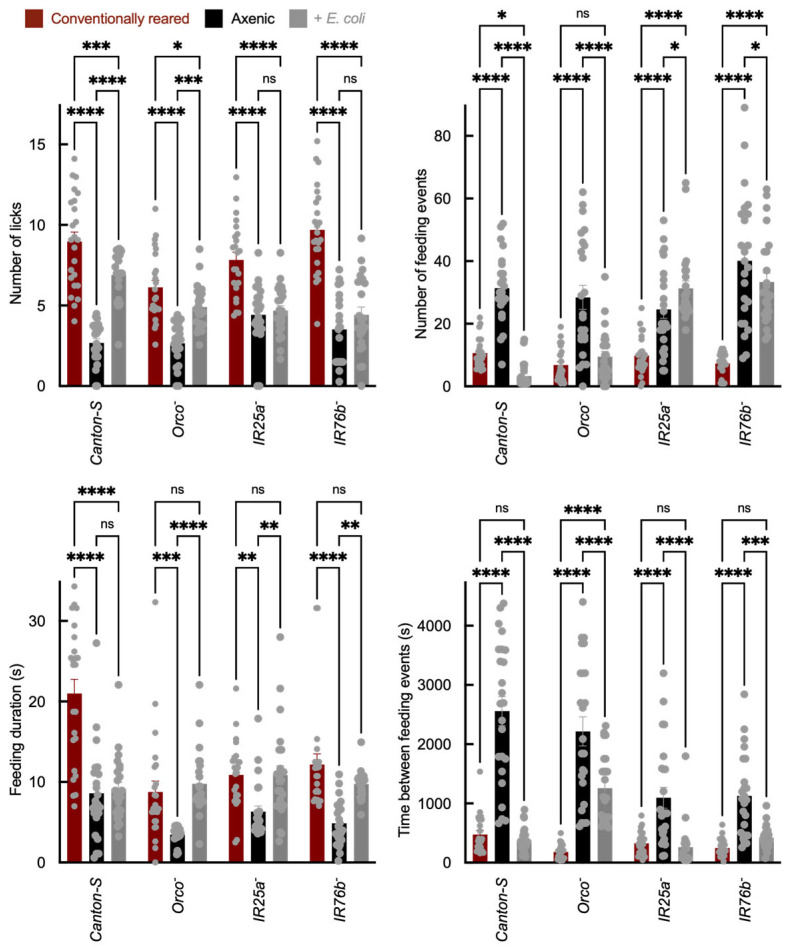
Sucrose feeding behavior measured using the Fly Liquid-Food Interaction Counter (FLIC). Sucrose feeding behavior measured using the FLIC system, showing total feeding signal and bout structure. The y-axis shows total feeding signal (arbitrary units derived from FLIC electrical signal calibration), and the x-axis shows genotype and treatment group. Feeding bout structure (duration and number of bouts) is quantified per fly. Each biological replicate consisted of a single adult fly in one FLIC well (*N* = 24 per genotype × treatment). Bar colors indicate microbial status: red = conventionally reared, black = axenic, and gray = *E. coli*-associated. Error bars = mean ± SEM. Statistical comparisons were performed using two-way ANOVA with genotype and microbial status as factors, including the genotype × microbial status interaction term. Planned post hoc comparisons were conducted using Šídák’s multiple comparisons test. Significance: * *p* < 0.05, ** *p* < 0.01, *** *p* < 0.001, **** *p* < 0.0001, ns: not significant.

Feeding behavior was assessed using the Fly Liquid-Food Interaction Counter (FLIC), which reports feeding-related interactions as a calibrated electrical signal (arbitrary units) rather than direct volumetric intake. “Feeding” is defined as electrical contact between the proboscis and a sucrose solution. “Licks” represent individual proboscis contacts, while “events” correspond to continuous feeding bouts. Compared to axenic flies, *E. coli* association increased total feeding signal in *Canton-S* and *Orco*^−^ adults, whereas this increase was reduced or absent in *IR25a*^−^ and *IR76b*^−^ mutants (Figure 3). In addition to changes in total feeding signal, *E. coli*-associated flies exhibited altered feeding dynamics, including shifts in bout structure consistent with increased feeding drive. Because FLIC outputs integrate sensory valuation, internal metabolic state, and locomotor activity, they do not distinguish between direct modulation of gustatory signaling and broader physiological effects. However, the attenuation of these changes in IR co-receptor mutants suggests that IR pathways contribute to the observed feeding phenotypes.

Similar microbiome-mediated modulation of feeding behavior has been reported in *Drosophila*, where commensal bacteria shape host appetite and food choice by modulating amino acid availability and metabolic signaling [45,50]. Recent work also shows that bacterial peptidoglycan (PGN), a conserved component of Gram-negative cell walls, can directly activate gustatory neurons in *Drosophila* through immune signaling pathways, thereby altering feeding behavior [51]. This suggests that the effects we observe may reflect responses to broadly conserved bacterial cues rather than *E. coli*-specific metabolites.

These findings are consistent with broader evidence that bacterial signals can influence feeding-related pathways across systems [52]. While our mono-association design isolates the contribution of *E. coli*, similar effects may arise from other Gram-negative commensals that share structural components such as PGN. Future comparative assays across taxa (e.g., *Acetobacter* and *Lactobacillus*) will clarify whether these behavioral responses are strain-specific or reflect a generalized bacterial influence on insect feeding. Our data support the conclusion that *E. coli* association alters feeding-related behavioral outputs in an IR co-receptor-dependent manner under these experimental conditions.

Microbial modulation of attraction to fermentation cues and feeding-related behaviors may influence foraging decisions and host–microbe interactions. Similar dynamics are observed in yeast-fly mutualisms, where microbial volatiles attract flies that subsequently disperse microbes to new substrates [53]. However, because our experiments were performed under controlled laboratory mono-association conditions, the ecological significance and generality of these effects remain to be tested in more complex microbial communities and environmental settings.

The aversive or reduced-attraction phenotype observed in axenic flies further suggests that microbiota contribute to maintaining normal odor valence. The incomplete restoration of attraction following *E. coli* mono-association indicates that a single bacterial species may not fully recapitulate the metabolic and sensory contributions of a complex microbial community. These results suggest that community-level metabolic interactions may be required to fully restore typical fermentation cue preference.

## 4. Discussion

Our comparisons across conventionally reared, axenic, and *E. coli* mono-associated flies demonstrate that microbial status broadly affects multiple sensory-driven behaviors. Axenic flies frequently diverge from conventionally reared controls, indicating that baseline behavioral valence is influenced by the presence of a complex microbiota. *E. coli* mono-association partially restores behavioral phenotypes towards conventionally reared levels, suggesting that combined effects of resident microbiota and environmental microbes contribute to behavioral regulation in natural settings. Several microbial effects are reduced or absent in IR co-receptor mutants, consistent with an IR co-receptor-dependent contribution to specific phenotypes (e.g., acid- or amine-related cue responses and thermosensory outputs), while other phenotypes are IR-independent and may reflect systemic influences.

Across assays, our results support a phenotype partition: (i) behaviors that are microbe-sensitive but largely IR-independent (e.g., phototaxis changes across genotypes), consistent with systemic arousal/physiology effects; and (ii) behaviors with IR co-receptor dependence (e.g., thermal responsiveness and fermentation cue attraction), consistent with microbial modulation influencing IR-mediated sensory valuation pathways. This partition provides a testable framework for future experiments that distinguish sensory neuron modulation from systemic mechanisms.

Across both larval and adult stages, *E. coli* mono-association produced effects on thermosensation, tunneling, and olfactory attraction that were attenuated in IR co-receptor mutants, while modulating phototaxis and mechanosensation via IR-independent pathways. Because these assays integrate sensory processing with internal physiological state, our conclusions are limited to behavioral outcomes and do not establish a direct sensory-neuronal mechanism. These patterns are consistent with at least two non-mutually exclusive mechanisms. First, microbial association may influence behavioral outputs by modulating IR-dependent sensory pathways involved in cue detection and valuation. Second, microbial association may shift systemic physiology (e.g., metabolic or immune state), indirectly influencing locomotion, arousal, and feeding drive [54,55,56]. Distinguishing direct sensory modulation from indirect physiological effects will require sensory neuron-specific manipulations and independent measures of locomotor capacity and metabolic state.

*Drosophila*-microbe associations range from transient carriage driven by continuous ingestion to stable persistence that is taxon- and strain-dependent [31]. In *D. melanogaster*, the gut microbiota is composed of a relatively limited but functionally influential community dominated by *Acetobacter* and *Lactobacillus* species [57,58]. Quantitative work has shown that colonization outcomes are probabilistic and vary between hosts even under controlled dosing [59], while other studies demonstrate that stable colonization exists but is strongly species-specific and context-dependent [60]. The fly gut additionally imposes strong physiological barriers, including regional acidity and immune control, that limit the survival of many ingested bacteria [54,58,61], reflecting a dynamic equilibrium between acquisition and loss rather than obligate, long-term colonization. Within this framework, our mono-association approach represents a controlled test of sufficiency under defined conditions and does not imply that *E. coli* uniquely drives these behaviors in natural settings. Furthermore, immune and physiological regulatory mechanisms in the fly gut, including reactive oxygen species production and antimicrobial peptide activity, actively shape microbial persistence and composition [62,63], reinforcing the fact that detection of a bacterium during an assay does not imply stable colonization without longitudinal evidence.

Because wild *Drosophila* are not axenic, any behavioral effect of a given bacterium in nature would occur within a resident microbial community and environmental context [31,64]. Our mono-association experiments, therefore, establish sufficiency under defined conditions and motivate future tests in poly-association or conventional microbiota contexts.

Studies of natural *Drosophila* populations show that gut microbiota vary across environments and host genotypes, indicating that the fly microbiome is diverse and dynamic in nature [65]. A growing body of work demonstrates that the *Drosophila* microbiome broadly modulates host behavior [51,66], including olfactory-guided foraging, nutrient preference, feeding, and social and reproductive behaviors. For example, gut microbiota composition shapes olfactory-guided microbial preferences and foraging trade-offs [67]; while commensal bacteria can buffer dietary amino acid imbalance, thereby altering food choice and reproductive output [45]. Beyond single-species effects, metabolite exchange among microbial community members can generate emergent volatile cues that drive attraction and oviposition-related decisions [25,68]. These findings support the interpretation that behaviorally relevant chemical ecology depends on microbial community context rather than any single microbe.

Our findings align with prior work showing that members of the genera *Lactobacillus* and *Acetobacter* modulate olfactory and gustatory behaviors by altering nutrient metabolism, odorant binding protein expression, and amino acid signaling [45,50,67,68]. Commensal bacteria also influence mating preferences and cuticular hydrocarbon profiles, reinforcing the bidirectional link between microbial metabolism and chemosensory communication [56,69]. While endosymbionts such as *Wolbachia* can alter transcription of olfactory receptors [70], free-living commensals provide ecologically relevant parallels to *E. coli*, which transiently associate with hosts and influence sensory-driven behavior through metabolite exchange. The conservation of IR co-receptors such as IR25a and IR76b across insect orders suggests that such microbe-mediated modulation of chemosensory pathways may represent a broadly conserved feature of insect-microbe interactions. Community effects arising from metabolic cross-talk (e.g., acetate, TCA intermediates) among taxa [71] further indicate that host behavioral traits can be shaped indirectly through microbial interactions.

In summary, microbial status exerts broad effects on *Drosophila* behavior across developmental stages. Under gnotobiotic conditions, *E. coli* mono-association alters specific sensory-driven outputs, with several effects attenuated in IR co-receptor mutants. Because wild flies harbor complex microbial communities, these findings should be interpreted within the broader ecological and community context of the fly microbiome. Future work should identify the bacterial signals and host pathways that underlie these behavioral changes.

## Data Availability

The original contributions presented in this study are included in the article/Supplementary Material. Further inquiries can be directed to the corresponding author.

## Supplementary Materials

The following supporting information can be downloaded at: https://www.mdpi.com/article/10.3390/insects17030275/s1, Figure S1: Axenic status confirmation of experimental fly lines by culture-based plating.

## Abbreviations

The following abbreviations are used in this manuscript:

ACV	Apple cider vinegar
FLIC	Fly Liquid-Food Interaction Counter
IR	Ionotropic receptor
LB	Luria broth
OR	Olfactory receptor
PI	Preference index
RH	Relative humidity
SD	Standard deviation
SEM	Standard error of the mean
