# Supplementary material for: Escherichia coli Mono-Association Modulates Ionotropic Receptor-Dependent Behaviors in Drosophila melanogaster
Source: Insects. 2026 Mar 3;17(3):275. doi: 10.3390/insects17030275 (PMC13026950; doi:10.3390/insects17030275)
Supplement: Supplementary file 1 [file insects-17-00275-s001.zip › insects-4115868-supplementary.pdf]

Supplementary Materials

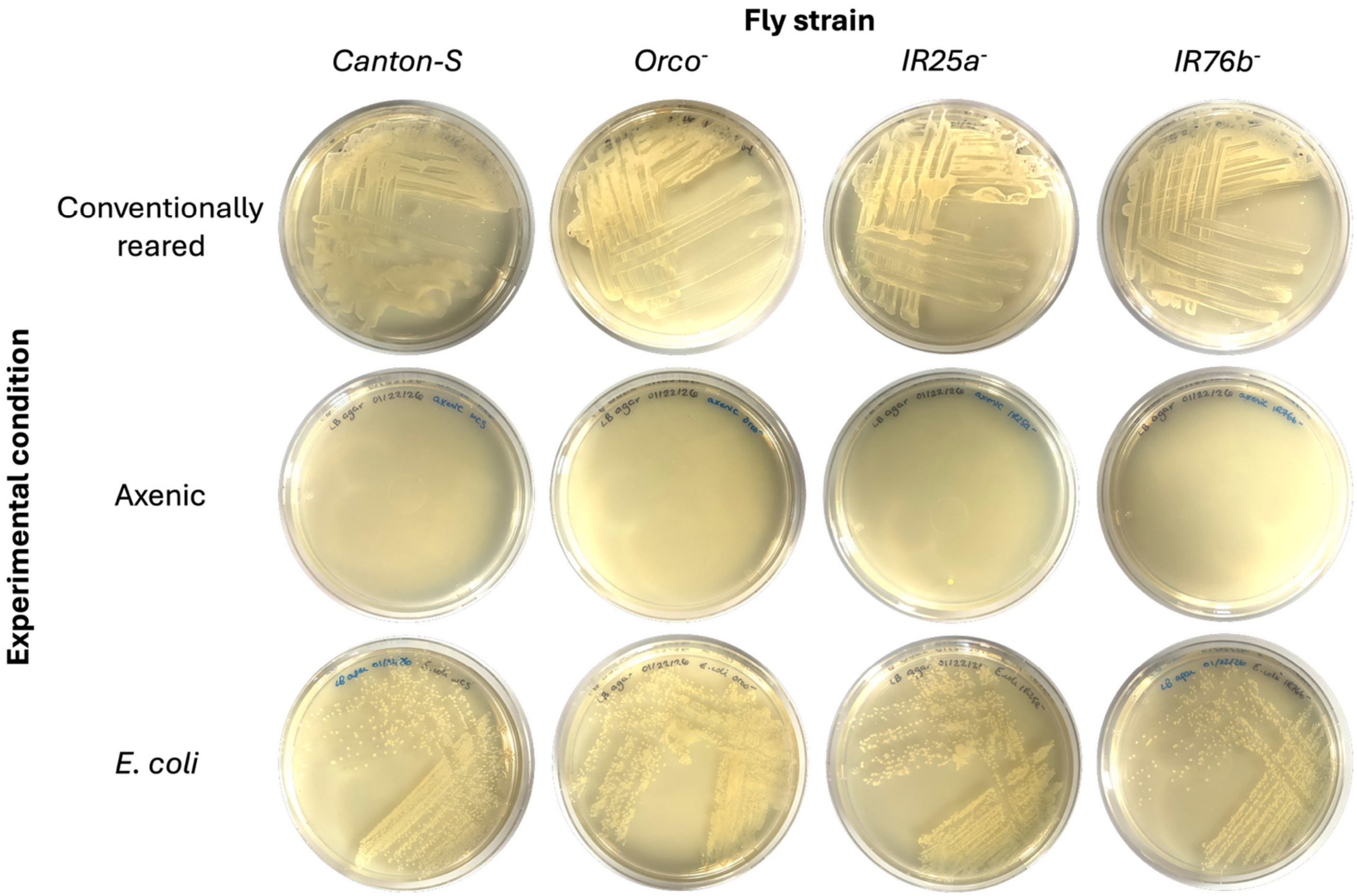

Figure S1. Axenic status confirmation of experimental fly lines by culture-based plating. Representative LB agar plates showing bacterial growth from homogenates of 10 surface-sterilized adults from experimental fly lines (*Canton-S*, *Orco*$^{-}$, *IR25a*$^{-}$, and *IR76b*$^{-}$) under three conditions. Top row: conventionally reared flies with their inherent microbiome intact. Middle row: axenic (germ-free) cohorts show no detectable colony growth. Bottom row: *E. coli*-recolonized cohorts show robust colony growth.
